# High-Bonding-Strength Polyimide Films Achieved via Thermal Management and Surface Activation

**DOI:** 10.3390/nano13091575

**Published:** 2023-05-08

**Authors:** Pin-Syuan He, Dinh-Phuc Tran, Ting-Yu Kuo, Wei-You Hsu, Huai-En Lin, Kai-Cheng Shie, Chih Chen

**Affiliations:** 1 Department of Materials Science and Engineering, National Yang Ming Chiao Tung University, Hsinchu 30010, Taiwan; syuan.en09@nycu.edu.tw (P.-S.H.); trandinhphuc1508@nycu.edu.tw (D.-P.T.); kuo24333.en09@nycu.edu.tw (T.-Y.K.); mice803.c@nycu.edu.tw (W.-Y.H.); williamlin.en09@nycu.edu.tw (H.-E.L.); alex911666.mse06g@g2.nctu.edu.tw (K.-C.S.); 2 Department of Materials Science and Engineering, National Chiao Tung University, Hsinchu 30010, Taiwan

**Keywords:** polyimide, bonding strength, partial curing, hybrid bonding, surface modification

## Abstract

In this study, thermal and argon (Ar) plasma/wetting treatments were combined to enhance the bonding strength of polyimide (PI) films. Attenuated total reflectance-Fourier transform infrared spectroscopy (ATR-FTIR) was used to analyze the changes in the PI imidization degrees. The contact angles of the PI films were also measured. The results show that the contact angles of the fully cured PI films markedly decreased from 78.54° to 26.05° after the Ar plasma treatments. X-ray photoelectron spectroscopy (XPS) analysis was also conducted on the PI surfaces. We found that the intensities of the C-OH and C-N-H bonds increased from 0% to 13% and 29% to 57%, respectively, after Ar plasma activation. Such increases in the C-OH and C-N-H intensities could be attributed to the generation of dangling bonds and the breakage of the imide ring or polymer long chains. Shear tests were also conducted to characterize the bonding strength of the PI films, which, after being treated with the appropriate parameters of temperature, plasma power, and wetting droplets, was found to be excellent at greater than 35.3 MPa.

## 1. Introduction

Currently, chips and wafers with different functions are manufactured separately and vertically stacked via hybrid bonding processes, which offer a promising solution for three-dimensional (3D) integration to continue Moore’s law [1,2,3]. The wafer-level hybrid bonding of Cu and silicon oxides has already been applied in the mass-production stacking of back-illuminated CMOS image sensors (BI-CIS) [4]. However, the hybrid bonding of inorganic dielectrics, such as PECVD SiO_2_ and SiCN, is very complicated and not cost-effective. In contrast, organic dielectrics are considered to be low-cost, and spin coating can be used to deposit a thin organic layer onto a substrate. Hybrid bonding can be cost-effectively achieved using organic dielectrics (polyimide (PI), polybenzoxazole (PBO), and benzocyclobutene (BCB)) due to their tunable properties [5,6,7,8]. It has been reported that PI possesses low dielectric constants (*D*_k_ = ~3.61 at 1 MHz and *D*_f_ = ~0.037 at 1 MHz), a low curing temperature (~230 °C), processing simplicity, strong mechanical properties, high electrical resistance, and great thermal stability [9]. It is considered to be a strong candidate for the passivation of the bonding interface and the enhancement of hybrid films’ bonding strength, and it is also commonly applied as a protection and stress-relief layer in the redistribution layers of 2.5D packaging. Thus, with its existing fabrication capabilities, PI is suitable for hybrid bonding. The curing of PI involves an imidization reaction. The transition between the solvent evaporation and imidization reaction is a key factor that improves the glass transition temperature (*T*_g_ = 210 °C, when it is fully cured) and the mechanical properties. The change of *T*_g_ with the degree of imidization (DI) is crucial and very complex during the imidization. The diffusion of the local polymer chains slows down below *T*_g_ [10], resulting in low molecular mobility. Thus, the *T*_g_ increases with an increasing imidization degree, and a high *T*_g_ can prevent further imidization.

In this study, attenuated total reflectance-Fourier transform infrared spectroscopy (ATR-FTIR) was used to analyze the PI imidization degree [11]. The correlations between heat treatment, various imidization degrees (from soft baking to the standard full curing of PI), bonding temperatures, and time were then investigated. To suppress the outgassing and misalignment issues in fine-pitch hybrid bonding, the standard full curing of PI bonding was also conducted. Moreover, argon (Ar) plasma surface modification and wetting treatments were applied to enhance the bonding strength and to reduce the bonding temperature and/or time required of the fully cured PI. This study is divided into three parts: (1) the various PI film imidization degrees with fixed bonding temperature and time, (2) the fixed imidization degree (fully cured) of the PI films with varying bonding temperatures and times, and (3) the fixed imidization degree (fully cured) of the PI films with Ar plasma surface and wetting modification.

## 2. Materials and Methods

**Figure 1:**
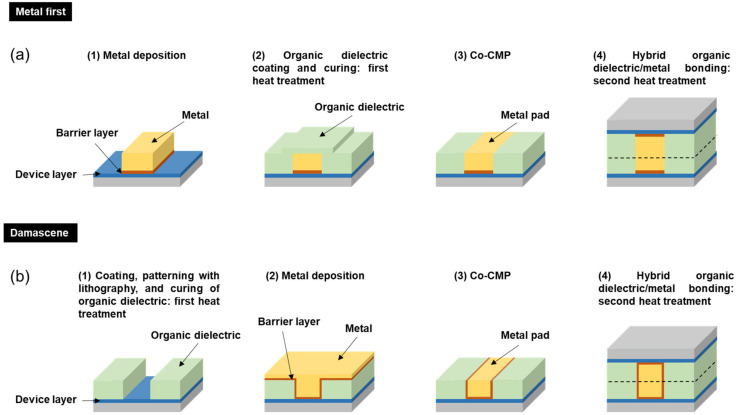
Schematic of the PI/metal hybrid bonding processes: (**a**) metal-first and (**b**) damascene.

In the semiconductor industry, the hybrid bonding processes consist of two heat treatment steps: PI curing and thermocompression bonding, as shown in Figure 1. The PI/metal hybrid bonding can be conducted using metal-first (Figure 1a) and damascene (Figure 1b) methods. To explore the various imidization degrees of PI films, a PI precursor was spin-coated on 8” wafers and cured under different curing conditions at 100 to 230 °C for 4 to 180 min. Chemical mechanical planarization (CMP) was then conducted to flatten the PI surfaces [12]. The surface roughness (*R*_q_) was approximately 1~2 nm after the CMP. The average PI thickness of the wafers was about 8~9 μm, with a variation of 0.2 μm. The specimens were then diced into two physical dimensions of 0.6 × 0.6 cm^2^ and 1.5 × 1.5 cm^2^ prior to bonding. Note that the PI (LTC9320 series, Fujifilm Electronic Materials, North Kingstown, RI, USA) used in this study had a low standard curing temperature of 230 °C, and the fully cured PI had a *T*_g_ of 210 °C [9]. Such material properties play crucial roles in the bonding process [10]. Through the use of PI films, the bonding temperature and time were expected to be reduced.

Prior to bonding, the top and bottom dies were cleaned using acetone, citric acid, and DI water. They were then transferred to a vacuum furnace. We also transferred several PI films after each partial curing to the vacuum furnace for the same heat treatment. The changes in the imidization degree between the two steps of the heat treatment were analyzed using an ATR-FTIR (Thermo Nicolet 5700 with Microscope Continuum, Thermo Scientific, Waltham, MA, USA) spectroscope. In the current study, we employed an internal standard method to calibrate the peak height of the ATR-FTIR spectrum to avoid errors. To compare the DI changes, the raw spectra were normalized via a reference peak; this is the internal standard component, which was the aromatic ether of PI [13] in this study, and its concentration was fixed in every process. We set its peak height to 1, and normalized the whole spectrum; thus, the contributions of different degrees of roughness and other errors could be eliminated.

During bonding, screw bonders were used to apply the bonding forces. The samples were bonded under different conditions: (1) 200 °C for 30 min, (2) 100 °C, 150 °C, 200 °C, and 250 °C for 30 min and 200 °C for 30 and 60 min, and (3) 200 °C for 30 min. To suppress the outgassing of the PI, a vacuum environment was maintained in the furnace [14]. In this study, we used Ar plasma and water droplets instead of O_2_ plasma to passivate the bonding surfaces of the fully cured PI films to enhance the bonding quality, because the O_2_ plasma treatment might have led to the serious oxidation of the metal pads in the ex-situ plasma-bonding process. The C−OH groups were multiplied on the bonding surfaces, leading to increases in the surface reactivity and hydrophilicity [15,16]. The Ar plasma power was tuned from 20 to 100 W. Water droplets in the volume of 1 μL were then spread on the specimens. They were finally transferred to screw bonders for the thermal compression bonding.

A contact angle meter (FTA-125, First Ten Angstroms, Newark, CA, USA) and a 0.1 μL water droplet were used to characterize the hydrophilicity of the PI surfaces. The surface roughness was measured using an atomic force microscope (AFM, Innova AFM, Bruker, Billerica, MA, USA). X-ray photoelectron spectroscopy (XPS, ESCALAB Xi+, Thermo Scientific, Waltham, MA, USA) combined with a micro-focused monochromatic Al *K*_a_ (h_v_ = 1486.6 eV) source was utilized to analyze the chemical states of the PI surfaces before and after the Ar plasma treatments. An energy step size (0.1 eV) was set, and the diameter of the X-ray beam was 650 μm. The numbers of the scans and energy steps were set as 15 and 231, respectively. Note that C is the main component in the PI; thus, the raw data were deconvoluted into different components, and then the C-C raw data were corrected, which were referred from the NIST inorganic crystal structure database (ICSD) and had a binding energy of 284.8 eV. The detailed guidelines for the XPS analysis can be found in previous studies [17,18,19,20,21,22,23]. The bonding interfaces were observed using a focused ion beam (FIB, FEI Helios G3CX, Thermo Fisher Scientific, Waltham, MA, USA). Scanning acoustic tomography (SAT, CSAM, Nordson SONOSCAN-Gen6, Elk Village, IL, USA) was also employed to observe the bonding area ratio. The bonding strength of the PI-PI films was measured using shear tests with a speed of 100 μm/s and a shear height of 200 μm.

## 3. Results

### 3.1. Imidization Degree Change in PI under Fixed Bonding Temperature and Time

#### 3.1.1. Imidization Degree of PI

**Figure 2:**
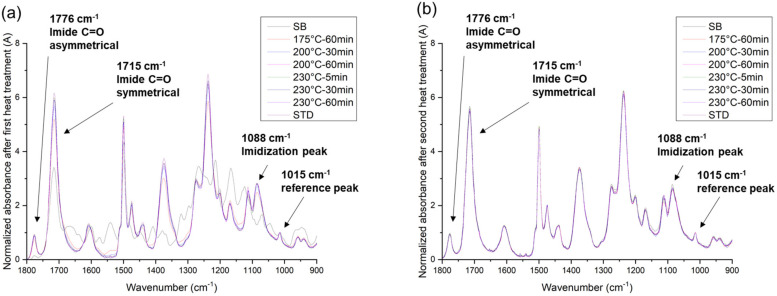
The ATR-FTIR normalized spectra of the PI films after the (**a**) first and (**b**) second heat treatments.

The curing process of the PI films correlated with the thermal imidization reaction, which underwent imide ring closure and intramolecular dehydration. In this study, we conducted the curing of the films under different partial curing conditions. ATR-FTIR analysis was also conducted to investigate the change in the imide group on the PI surfaces. From the normalized profiles of the ATR-FTIR spectra, the DI values after the first and second heat treatments were obtained as follows:

(1) $$DI\;\left(\%\right)=\frac{{\left(\frac{A_{I}}{A_{s}}\right)}_{partial\;curing}-{\left(\frac{A_{I}}{A_{s}}\right)}_{soft\;bake}}{{\left(\frac{A_{I}}{A_{s}}\right)}_{full\;curing}-{\left(\frac{A_{I}}{A_{s}}\right)}_{soft\;bake}}$$

where *A*_I_ and *A*_S_ are the imidization and reference peak heights, respectively. The imidization peak was at 1088 cm^−1^, and the reference peak, which represents the internal standard component (aromatic ether) in the PI structure, was at 1015 cm^−1^. The imidization degree of the soft-baked PI was defined as 0%, and the fully cured PI was defined as 100% in the above equation [24]. The normalized ATR-FTIR spectra of the PI films after the first and second heat treatments are shown in Figure 2. According to the normalized imide peak after the first heat treatment (PI curing, Figure 2a), we found that the DI value was more sensitive to the temperature than to the curing time [25,26]. This could be attributed to the fact that the thermal imidization of PI is strongly dependent on the evaporation of its polar aprotic solvent [10]. As the partial curing temperature increased to the boiling point of the PI solvent (~204 °C), the imidization reaction accelerated, resulting in a high DI value. The normalized imide peak after the second heat treatment at 200 °C for 30 min is shown in Figure 2b. It can be seen that all of the DI values reached almost 100%, no matter what the initial partial curing conditions were. This indicates that the PI films were fully cured after the second heat treatments.

**Figure 3:**
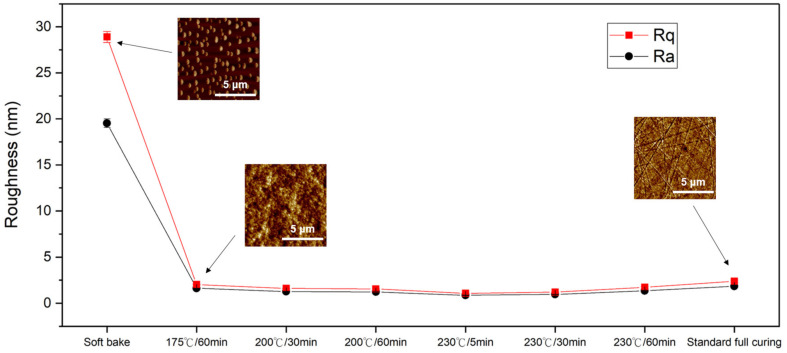
Surface roughness of the PI films under different curing conditions. Typical AFM images are also presented.

Surface roughness is one of the main factors affecting the bonding quality [27]. Figure 3 shows the correlation between different imidization degrees and the surface roughness of the PI. Some typical AFM images are also presented in Figure 3. Note that we conducted CMP on the PI films after curing and measured their surface roughness. The root mean square roughness (*R*_q_) of the fully cured PI films was ~2.39 nm. It can be seen that the roughness of the soft-baked PI significantly increased after the CMP because such films were soft and easy to remove. The roughness slightly increased as the curing temperature and/or time decreased in accordance with the decrease in the imidization of the PI films (Figure 2).

#### 3.1.2. PI-PI Bonding under Fixed Temperature and Time

**Figure 4:**
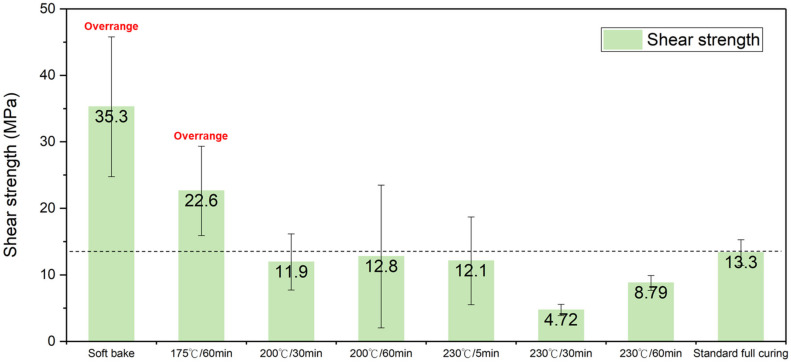
Shear strength of the PI-PI films bonded under different curing conditions.

**Figure 5:**
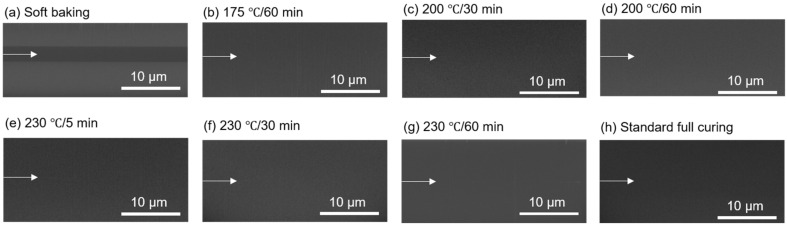
Cross sectional FIB e-beam images of the PI-PI films bonded under different curing conditions. The white arrows indicate the eliminated bonding interfaces: (**a**) soft baking, (**b**) 175 °C/60 min, (**c**) 200 °C/30 min, (**d**) 200 °C/60 min, (**e**) 230 °C/5 min, (**f**) 230 °C/30 min, (**g**) 230 °C/60 min, and (**h**) standard full curing.

**Figure 6:**
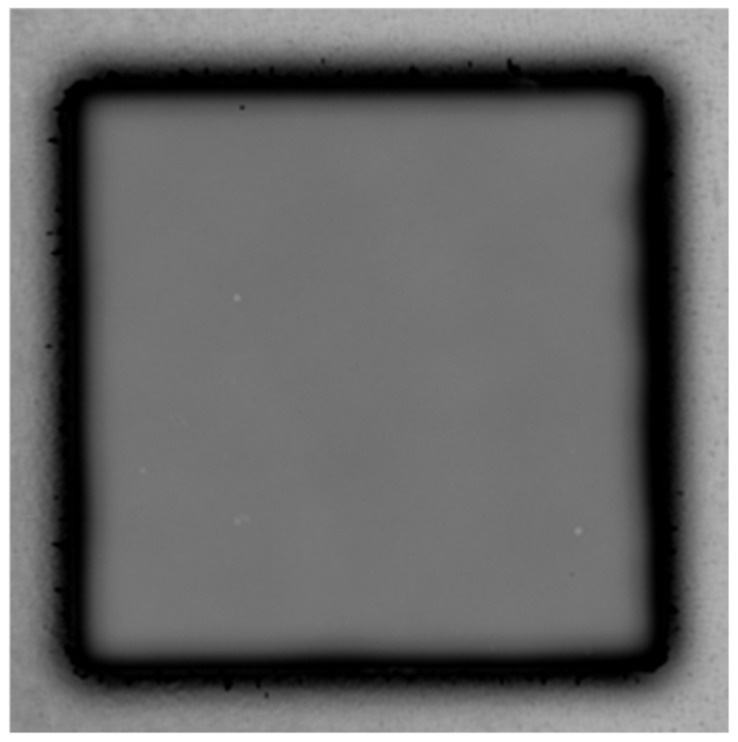
SAT image of the standard fully cured PI-PI bonded films.

The shear strength of the PI-PI films with various imidization degrees, which bonded at the same bonding temperature (200 °C) and time (30 min), is shown in Figure 4. As the films were cured at a temperature below 200 °C, which was near the boiling point of the PI solvent, the bonding strength significantly increased (>35.3 MPa). It even surpassed the detection limit of the shear testing machine. Thus, the true bonding strength could be greater than 35.3 MPa. The cross-sectional FIB e-beam images of the PI-PI films are shown in Figure 5. It can be observed that the bonding interfaces were eliminated under all curing conditions. A typical SAT image of the fully cured PI-PI films is shown in Figure 6. A strong bonding quality without obvious defects of such films could be achieved.

### 3.2. Fixed Imidization Degree (Fully Cured) of the PI under Different Bonding Temperatures and Times

#### PI-PI Bonding under Different Temperatures and Time

**Figure 7:**
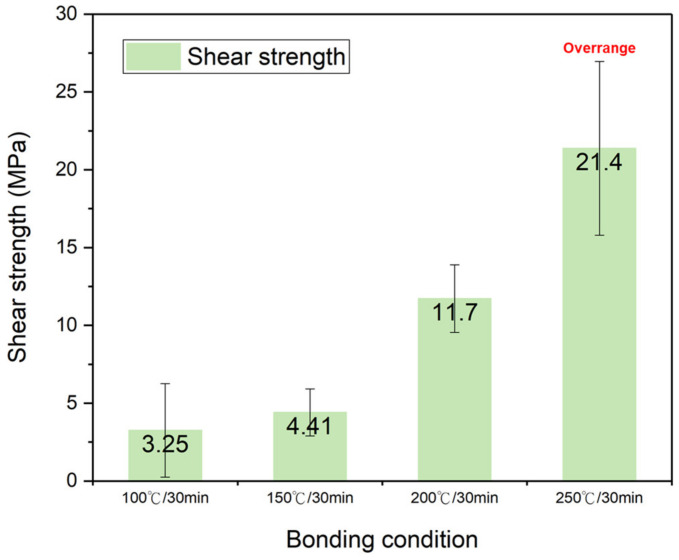
Bonding strength of the fully cured PI-PI films bonded at different temperatures.

**Figure 8:**
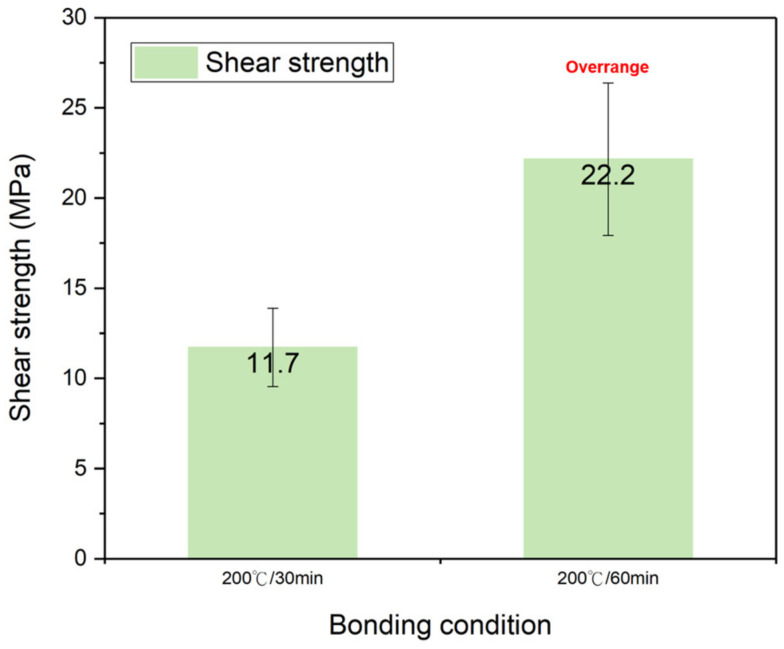
Bonding strength of the fully cured PI-PI films bonded for different durations of time.

The shear strength of the fully cured PI-PI films bonded at different bonding temperatures of 100 °C, 150 °C, 200 °C, and 250 °C for 30 min is shown in Figure 7. The fully cured PI films were chosen to analyze the effects of the bonding temperature and time. When the bonding time was fixed, the bonding strength of the films increased with the increasing bonding temperature. A high bonding strength of 21.4 MPa was achieved, when they bonded at 250 °C for 30 min. This was equivalent to the bonding strength of the films bonded under a partial curing condition (175 °C for 60 min), as aforementioned. The shear strength of the fully cured PI-PI films bonded at 200 °C for 30 min and 60 min is shown in Figure 8. When they bonded at a similar temperature (200 °C), the bonding strength increased when the bonding time increased. Figure 7 and Figure 8 clarify the effects of the bonding temperature and time. When the bonding time doubled, the bonding strength increased by ~1.9 times, and when the bonding temperature increased by two times, the bonding strength increased by ~3.6 times. It can be concluded that the PI-PI bonding strength was more sensitive to the bonding temperature rather than the time. Such a phenomenon could be attributed to the relation between the bonding temperature and the glass transition temperature (*T*_g_) of the PI films. Under the appropriate bonding condition, the local carbon chains were activated and moved to achieve bonding [28].

### 3.3. Fixed Imidization Degree (Fully Cured) of the PI with Ar Plasma Surface Modification and Wetting Treatment

#### 3.3.1. Effect of the Ar Plasma on the PI Surfaces

**Figure 9:**
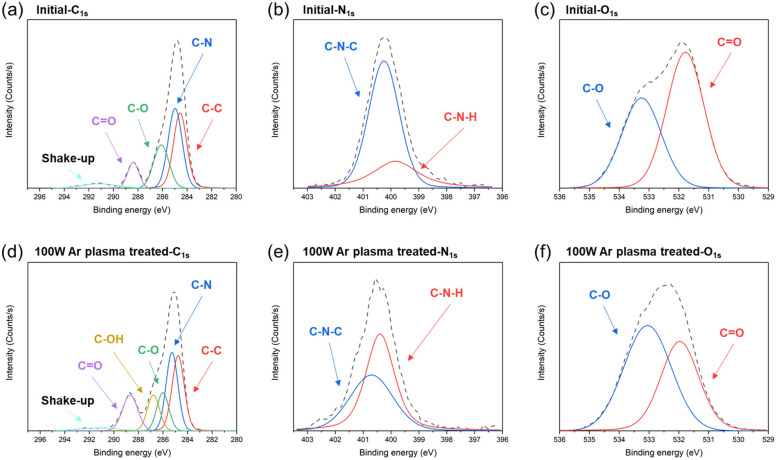
XPS spectra of the PI films (**a**–**c**) before and (**d**–**f**) after the Ar plasma treatments. The black dotted lines are denoted as the raw XPS spectra.

In this study, XPS was used to analyze the changes in the elements and chemical bonds of the fully cured PI surfaces before and after 100 W/2 min Ar plasma activation. The C 1s, N 1s, and O 1s spectra of the PI films before and after the plasma activation are shown in Figure 9. The C 1s spectrum (Figure 9a) was deconvoluted into five components: C-C at 284.8 eV, C-N at 285.3 eV, C-O at 286.1 eV, imide carbonyl C=O at 288.6 eV, and the shake-up peak at 291.1 eV. The N 1s spectrum (Figure 9b) was deconvoluted into two components: imide C-N-C at 400.7 eV and amine C-N-H at 399.8 eV. The O 1s spectrum (Figure 9c) was deconvoluted into two components: C-O at 533.2 eV and C=O at 531.8 eV. The XPS spectra of the PI films after being treated with 100 W Ar plasma were revealed by the element spectra. As shown in the C 1s spectra (Figure 9a,d), the intensities of the C-C, C-N, and C-O bonds slightly decreased, while the C=O intensity increased after Ar plasma activation. Additionally, we found a new hydrophilic group of C-OH bonds (at 287 eV). Compared with the N 1s spectra (Figure 9b,e), the intensity of the imide C-N-C bonds markedly decreased from 71% to 43%, and that of the C-N-H bonds greatly increased from 29% to 57%. As shown in the O 1s spectra (Figure 9c,f), the intensity of the C-O bonds increased from 41% to 58%, and the C=O decreased from 59% to 42%. These XPS results indicate that the Ar plasma could break the bonds of the imide rings and long polymer chains on the PI surfaces into short-chain molecules and simultaneously generate hydrophilic C-OH groups. The PI surfaces were thus more hydrophilic [29,30,31], which was beneficial for the bonding of the PI films.

**Figure 10:**
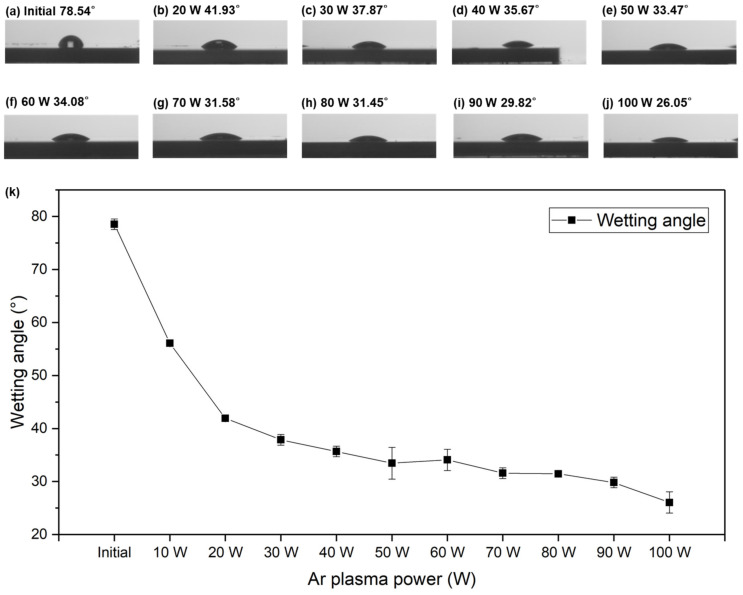
Wetting angles of the PI films (**a**) before and after exposure with (**b**) 20 W, (**c**) 30 W, (**d**) 40 W, (**e**) 50 W, (**f**) 60 W, (**g**) 70 W, (**h**) 80 W, (**i**) 90 W, and (**j**) 100 W of Ar plasma. (**k**) Correlation between the wetting angle and the Ar plasma power.

**Figure 11:**
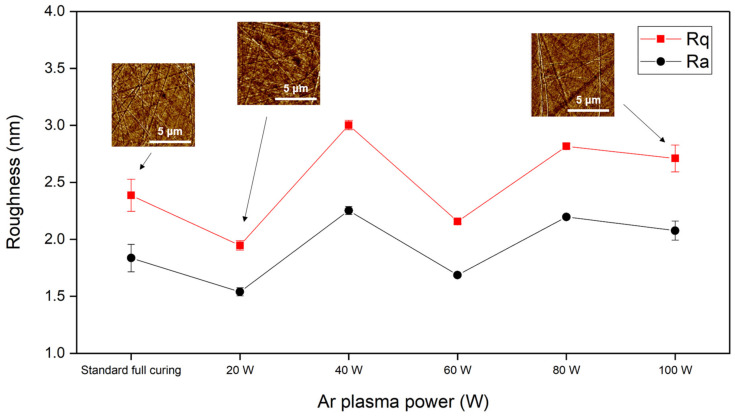
Surface roughness of the PI films before and after the Ar plasma treatments.

In addition to the XPS analysis, the effect of the Ar plasma treatments on the hydrophilicity of the PI films was also characterized by measuring their wetting angles. Figure 10 shows the wetting angles of the fully cured PI surfaces before and after exposure to different powers of Ar plasma for 2 min. The wetting angle decreased from 78.54 to 26.05°, and the wetting angles and the Ar plasma powers had a negative correlation. These results indicate that the PI surfaces were more hydrophilic after the Ar plasma treatments. However, the etchings left by the Ar plasma bombardment could be of concern. Thus, the surface roughness was also measured in this study. Figure 11 shows the AFM images and surface roughness of the PI films before and after Ar plasma exposure for 2 min. Note that the roughness of the films was found via AFM-measuring at three different regions in each PI sample (0.6 × 0.6 cm^2^), and the scan area was 10 × 10 μm^2^. Figure 11 shows that the standard deviations were very small. The possible factors causing such fluctuations could be attributed to the CMP process, the selection of the AFM area, the reliability of the plasma machine, and the ex-situ plasma process. The plasma treatments did not cause serious ion bombardment issues or increased the amplitude and/or number of hillocks obviously in current study [20]. After the 20 W Ar plasma treatment, the surface roughness (*R*_q_) of the fully cured PI slightly decreased. However, it slightly increased when the Ar plasma power was higher than 30 W. Such slight changes in the surface roughness were still acceptable in achieving a good bonding quality.

#### 3.3.2. PI-PI Bonding with the Ar Plasma Surface Modification and Wetting Treatment

**Figure 12:**
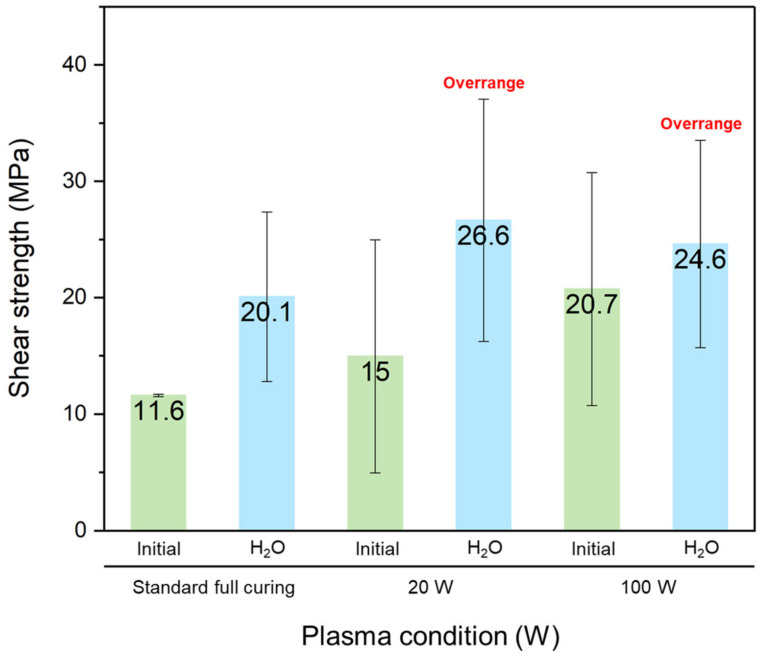
Shear strength of the PI-PI films treated with the Ar plasma and water-wetting droplets.

In addition to the Ar plasma activation, we also conducted water-wetting treatments on the PI films prior to bonding. The shear strength of the PI-PI films treated with different degrees of Ar plasma power for 2 min and 1 μL water droplets is shown in Figure 12. The films were bonded at the same bonding temperature of 200 °C for 30 min. It is obvious that the plasma treatments were beneficial for the bonding strength of the PI-PI films. The wetting treatments further assisted the Ar plasma in enhancing the bonding strength.

## 4. Discussion

As mentioned in Section 3.1 and Section 3.2, it can be concluded that the curing/bonding temperature was more effective than the curing/bonding time. Furthermore, the lower the imidization degree of PI, the higher the bonding strength of the PI-PI films that could be achieved. We found that the surface roughness of the soft-baked PI films was greater than that of the other cured films. The bonding strength of the soft-baked films was the highest, which was contrary to the metal–metal bonding [32]. This could be attributed to the thermal imidization reaction and the glass transition temperature of the PI films. If the imidization degree was lower than 100% after the first heat treatment, the imide ring could still close and rearrange the molecular chains because of the van der Waals force. Thus, the bonding strength of the PI films increased. The other reason could be the glass transition temperature (*T*_g_), which has been reported to change with the imidization degree [10], whereby the PI molecules are more active above *T*_g_ [28]; thus, the PI molecular chains could diffuse across the bonding interface. This could be progressively eliminated when the bonding temperature reached *T*_g_, leading to a great bonding strength (>35.3 MPa). However, with regard to the hybrid bonding process, there were other important factors that needed to be considered besides the high bonding strength. During thermal imidization, the evaporation of the PI solvent and imide ring closure would cause volume shrinkage and other property issues in relation to the PI films. If we take soft baking as an example, although the high bonding strength could be achieved due to the first heat treatment condition, the issue might appear during the co-CMP process, as shown in Figure 1. It is noted that the soft-baked PI possessed the worst chemical resistance and had weak mechanical properties. It was also difficult to control the thickness removal (as it might have been over-polished). Additionally, it was easy for the films to be peeled off during the CMP process. The leftover PI solvent and/or gas might have been trapped in the bulk PI and caused some reliability issues for the hybrid joints.

In addition, after the Ar plasma activation and wetting treatments, new hydrophilic OH groups were detected on the PI surfaces. The XPS results (Figure 9) showed that the Ar plasma tended to break the C-C, imide C-N, and C=O bonds, which was beneficial for the formation of C-O and amine C-N-H groups on the PI surfaces. The Ar plasma also induced low-density hydrophilic groups (C-OH) on the PI surfaces and thus effectively enhanced the adsorption of water molecules on the PI surfaces. This resulted in a large number of hydrophilic OH groups. The adsorbed water molecules thus facilitated the pre-bonding at the bonding interfaces [20,33]. Moreover, the considerable number of hydrophilic groups on the surfaces induced various bridging bonds between the PI films, which led to the compact contact of atoms. The hydration reaction then triggered the generation of newly stable covalent bonds under appropriate bonding conditions. Thus, an enhancement in the bonding quality could be achieved. Such a phenomenon can be considered as the synergistic effect of the Ar plasma and water droplets. The combined effect of the plasma activation and wetting treatments on the PI films resulted in the bonding strength of the PI films being high. Such high-strength PI films can be used for hybrid dielectric bonding in fine-pitch 3D integration with a low thermal budget and low cost.

## 5. Conclusions

In summary, PI films were cured and bonded under different conditions. The changes in the PI imidization degrees were characterized using ATR-FTIR. The PI bonding mechanism was analyzed and correlated with the thermal imidization reaction and glass transition temperature. We also incorporated Ar plasma and wetting droplets to enhance the bonding strength of the fully cured PI films. The results show that the contact angles of the fully cured PI films significantly reduced from 78.54° to 26.05° after Ar plasma activation. Additionally, XPS analysis was conducted on the PI surfaces. We found increases in the C-OH (0% to 13%) and C-N-H (29% to 57%) intensities and a decrease in the imide C-N-C peak after plasma activation. Such increases in the C-OH and C-N-H intensities could be attributed to the generation of dangling bonds and the breakage of the imide ring and/or polymer long chains, which enhanced the further hydration bridging. These findings could be beneficial for the generation of stable covalent bonds under appropriate bonding conditions. The microstructural analysis indicated that all PI bonding interfaces were eliminated, resulting in a high bonding strength (>35.3 MPa) of the PI films.

## Data Availability

Data available upon request due to restrictions, e.g., privacy or ethical.
